# Comprehensive NGS Panel Validation for the Identification of Actionable Alterations in Adult Solid Tumors

**DOI:** 10.3390/jpm11050360

**Published:** 2021-04-29

**Authors:** Paula Martínez-Fernández, Patricia Pose, Raquel Dolz-Gaitón, Arantxa García, Inmaculada Trigo-Sánchez, Enrique Rodríguez-Zarco, MJose Garcia-Ruiz, Ibon Barba, Marta Izquierdo-García, Jennifer Valero-Garcia, Carlos Ruiz, Marián Lázaro, Paula Carbonell, Pablo Gargallo, Carlos Méndez, Juan José Ríos-Martín, Alberto Palmeiro-Uriach, Natalia Camarasa-Lillo, Jerónimo Forteza-Vila, Inés Calabria

**Affiliations:** 1 Imegen-Health in Code Group, 46980 Paterna, Spain; paula.martinez@imegen.es (P.M.-F.); mariajose.garcia@imegen.es (M.G.-R.); ibon.barba@imegen.es (I.B.); marta.izquierdo@imegen.es (M.I.-G.); jennifer.valero@imegen.es (J.V.-G.); carlos.ruiz@imegen.es (C.R.); marian.lazaro@imegen.es (M.L.); paula.carbonell@imegen.es (P.C.); pablo.gargallo@imegen.es (P.G.); 2 Servicio de Anatomía Patológica, Hospital Universitario de la Ribera, 46600 Alcira, Spain; p.pose14@gmail.com (P.P.); rdolzgaiton@gmail.com (R.D.-G.); 3 Servicio de Genética Molecular y Radiobiología, Centro Oncológico de Galicia, 15009 A Coruña, Spain; arantxa.garcia@cog.es; 4 Servicio de Anatomía Patológica, Hospital Universitario Virgen Macarena, 41009 Sevilla, Spain; intrigo@us.es (I.T.-S.); enriquerodriguezzarco@gmail.com (E.R.-Z.); jjrios@us.es (J.J.R.-M.); 5 Servicio de Oncología Médica, Centro Oncológico de Galicia, 15009 A Coruña, Spain; carlos.mendez@cog.es; 6 Laboratorio de Anatomía Patológica, Hospital General Universitario de Castellón, 12004 Castellón, Spain; a.alpaur@gmail.com; 7 Anatomía Patológica, Hospital Lluís Alcanyís de Xàtiva, 46800 Xàtiva, Spain; ncamarasalillo@gmail.com; 8 Anatomía Patológica, Universidade de Santiago de Compostela, 15705 Santiago de Compostela, Spain; jeronimo.forteza@gmail.com

**Keywords:** next-generation sequencing (NGS), validation, gene panels, actionable mutations, precision oncology, targeted therapies, adult solid tumors

## Abstract

The increasing identification of driver oncogenic alterations and progress of targeted therapies addresses the need of comprehensive alternatives to standard molecular methods. The translation into clinical practice of next-generation sequencing (NGS) panels is actually challenged by the compliance of high quality standards for clinical accreditation. Herein, we present the analytical and clinical feasibility study of a hybridization capture-based NGS panel (Action OncoKitDx) for the analysis of somatic mutations, copy number variants (CNVs), fusions, pharmacogenetic SNPs and Microsatellite Instability (MSI) determination in formalin-fixed paraffin-embedded (FFPE) tumor samples. A total of 64 samples were submitted to extensive analytical validation for the identification of previously known variants. An additional set of 166 tumor and patient-matched normal samples were sequenced to assess the clinical utility of the assay across different tumor types. The panel demonstrated good specificity, sensitivity, reproducibility, and repeatability for the identification of all biomarkers analyzed and the 5% limit of detection set was validated. Among the clinical cohorts, the assay revealed pathogenic genomic alterations in 97% of patient cases, and in 82.7%, at least one clinically relevant variant was detected. The validation of accuracy and robustness of this assay supports the Action OncoKitDx’s utility in adult solid tumors.

## 1. Introduction

Cancer can rely on different genetic aberrations, including point mutations, focal or complete chromosomal copy number variants, rearrangements or structural variants. Although some of them are well understood, the growing knowledge on less frequent pathogenic variants constituting driver events in tumorigenesis, together with the rapid development of targeted therapies, urges clinical testing to provide broader strategies [1].

Traditional molecular diagnosis based on low-throughput techniques for the detection of single nucleotide or small deletions and insertions is now being replaced by the pressing need of personalized medicine. Given the heterogeneity and genetic complexity that many tumors present, the integration of clinical, pathological, and molecular data provides key information to outline a specific biological profile that could be crucial in clinical decision-making. The current availability and cost-effectiveness of massively parallel next-generation sequencing (NGS) and the improvement of bioinformatic data analysis programs have enabled the implementation to the clinical practice of high-throughput, sensitive, and accurate tumor profiling [2].

Here we present the analytical validation and clinical utility of the Action OncoKitDx, a hybrid capture NGS-based panel indicated in the study of adult solid tumors. The Action OncoKitDx allows, through a single analysis, a comprehensive approach of the main actionable biomarkers relevant to oncological diagnosis, prognosis, and therapy. Furthermore, it includes the detection of somatic variants that represent eligibility criteria for the inclusion in several ongoing clinical trials.

This NGS panel integrates the complete sequencing of the 50 most relevant genes in adult cancer, the analysis of gene fusions and rearrangements related to targeted therapies, the detection of copy number variants (CNVs) throughout the genome, the analysis of microsatellite instability (MSI), and the pharmacogenetics associated with toxicity or efficacy of treatments used in oncology, allowing to obtain information of great utility in clinical practice.

Here we demonstrate the sensitivity, specificity, accuracy, and reproducibility of the Action OncoKitDx for the detection of clinically actionable variants.

## 2. Materials and Methods

### 2.1. Panel Design

The panel targets all tumor suppressors or oncogenes currently included in the standard of care of clinical diagnosis laboratories for a wide range of adult tumoral types. Besides including the main actionable biomarkers relevant for the therapy, prognosis, diagnosis, and resistance monitoring of cancer, determined by the National Comprehensive Cancer Network (NCCN, https://www.nccn.org/, accessed on 24 March 2021) and Spanish Society of Medical Oncology (SEOM, https://seom.org/, accessed on 24 March 2021) guidelines or related to approved treatments by international organizations as the Food & Drug Administration (FDA; https://www.fda.gov/, accessed on 24 March 2021) and the European Medicines Agency (EMA; https://www.ema.europa.eu/, accessed on 24 March 2021), it also interrogates many other genes related to tumorigenesis and potential targets for therapies being tested in clinical trials or susceptible to be developed in the future. 

The complete sequencing of the codifying region of a total of 50 genes identifies point mutations, including single-nucleotide variants and small indels, among *AKT1*, *AKT2*, *AKT3*, *ALK*, *ARID1A*, *ATRX*, *BRAF*, *BRCA1*, *BRCA2*, *CDH1*, *CTNNB1*, *EGFR*, *ERBB2*/*HER2*, *ESR1*, *ESR2*, *FGFR1*, *FGFR2*, *FGFR3*, *FGFR4*, *GNA11*, *GNAQ*, *HIST1H3H*, *HRAS*, *IDH1*, *IDH2*, *KIT*, *KRAS*, *MAP2K1*, *MET*, *MTOR*, *MYC*, *MYCN*, *NRAS*, *NTRK1*, *NTRK2*, *NTRK3*, *PDGFRA*, *PIK3CA*, *PBRM1*, *PMS2*, *PTEN*, *POLD1*, *POLE*, *RET*, *ROS1*, *SDHA*, *SDHB*, *TERT* (promoter included), *TP53,* and *VHL*. The detection of CNVs throughout the genome, from gains and losses of chromosomes or complete chromosomal arms, to specific genes and even exons, relies on three approaches: coverage of the 50 target genes, 500 SNPs distributed across the genome and off-target readings. It also includes the detection of fusion genes and breakpoint identification on selected intronic regions of *ALK*, *BCR*, *BRAF*, *EGFR*, *NTRK1*, *NTRK2*, *ETV6*, *RET*, and *ROS1* with any other partner of the genome. The Action OncoKitDx also allows the determination of MSI by the analysis of 110 markers, and pharmacogenetics associated with toxicity or efficacy of treatments used in oncology by analyzing ten high-evidence SNPs according to PharmGKB.

### 2.2. Sample Selection

For evaluation of the analytical performance of the assay, samples previously or subsequently analyzed with different methodologies and reference materials from Coriell Cell Repositories and Horizon Dx were also screened with the Action OncoKitDx. A total of 64 samples from different formalin-fixed paraffin-embedded (FFPE) tumor specimens, fresh frozen tissue, and peripheral blood were employed. Sensibility and specificity were assessed in 10 samples for point mutation and small indel detection, 10 samples for CNVs, 5 samples for fusions and large rearrangements, 6 samples for pharmacogenetics, and 45 samples for microsatellite instability evaluation. Both the pharmacogenetic and the CNV analyses used samples from previous sections. Likewise, the repeatability and reproducibility evaluation were carried out with samples previously analyzed in this validation.

To evaluate the assay’s performance in clinical specimens, tumor and patient-matched control samples from some of the most frequent adult solid cancer types were screened for mutations, including breast cancer, colorectal cancer, gastrointestinal stromal tumor, lung cancer, melanoma, ovarian cancer, pancreatic cancer, and other tumor types (endometrium, cervix, esophagus, larynx, prostate, kidney, thyroid, and bladder). Specifically, 138 tumor samples and 28 controls selected from different Spanish hospitals were included in this validation.

### 2.3. DNA Extraction and Quality Control

Selected samples were submitted to a pathological review in order to estimate the tumor cellularity. Genomic DNA (gDNA) from tumor and control tissue was extracted using the commercial extraction kits RecoverAll™ Total Nucleic Acid Isolation Kit for FFPE (Thermo Fisher Scientific, Waltham, MA, USA) and the QIAamp DNA Investigator Kit (Qiagen). Concentration was measured by fluorometric quantification using Qubit Fluorimeter (Thermo Fisher Scientific) with Qubit dsDNA BR Assay kit and Qubit dsDNA HS Assay kit (Invitrogen). DNA Integrity Number (DIN) was determined using the DNA ScreenTape assay (Agilent Technologies). Despite the recommended cut-off DIN value of 3 by the Action OncoKitDx user guide, samples with a DIN value above 2 were considered.

### 2.4. Library Preparation and Next-Generation Sequencing

The custom panel uses the SureSelect XT HS kit (Agilent), a commercial kit specially designed for small amounts of FFPE DNA as input and detect low allelic frequencies. After the mechanic fragmentation of 50–200 ng of tumoral gDNA from each sample to an average of 200 bases using the ME220 Focused-ultrasonicatorTM (Covaris), library preparation followed the Action OncoKitDx user guide. For the obtention of indexed libraries by molecular barcoding, DNA fragment ends were repaired and adenylated. Ligation of adapters incorporated unique molecular indexes (UMIs), specific DNA sequences that allow discrimination of each dsDNA molecule. Agencourt AMPure XP beads (Beckman Coulter) that selectively bind DNA 100 bp and larger were used for purification. Libraries were universally amplified by PCR (10–16 cycles, depending on the quality and amount of input DNA) with adapter-specific primers for the union of indexes, universal indexes, and sequencing adapters. Indexes are 8-nucleotide unique sequences compatible with Illumina adapters. They mark each sample’s library providing a unique combination, together with the universal indexes, that will allow bioinformatic analysis after sequencing. Indexed DNA fragments were then purified. Library quality control was performed on Tapestation 2000 and the commercial kits D1000 reagents and D1000 ScreenTape (Agilent). A mix of biotinylated capture probes specifically designed for the Action OncoKitDx were hybridized to regions of interest and then captured with streptavidin beads. Library enrichment involved the post-capture PCR amplification (12 cycles) and purification of products with Agencourt AMPure XP beads (Beckman Coulter). Libraries were validated and quantified using Qubit 2.0 fluorometer, Qubit ds DNA HS Assay kit (Invitrogen). A denaturation protocol was carried out prior to sequencing, using the NextSeq 500/550 Mid Output v2.5 and NextSeq 500/550 High Output v2.5 sequencing kits (Illumina) and Phix control (Illumina), and libraries were diluted to 1.5 pM. Pools were then loaded into the NextSeq 550 system (Illumina) for massive library sequencing in “Stand-alone” mode with 2 × 75 paired-end reads following the manufacturer’s instructions.

### 2.5. Bioinformatic Pipeline

The Sample Sheet necessary for sequencing was generated using the Illumina Experiment Manager (IEM) software version 1.14.0 (Illumina). Molecular barcoding allowed the elimination of optical (sequencing) and PCR duplicates without removing duplicates from different DNA molecules. The monitorization of sequencing run quality was based on the Q30 value and cluster pass filter, expecting measures greater than 80% and 70% respectively. The FASTQ files generated followed a quality evaluation applying the FastQC v0.11.5 software (Babraham Bioinformatics), measuring parameters such as the average quality per position of all readings, percentage of duplicate reads, sequence repeats patterns, etc. QC metrics were also evaluated from the BAM file of each sample in terms of uniformity, average coverage, and percentage of the region covered at 100× Bioinformatic analysis, including the alignment to the reference sequence Genome Reference Consortium Human Build 37 (GRCh37), annotation and variant calling, followed a self-developed pipeline through the DataGenomics platform. 

For the CNV analysis, in-house scripts were used to obtain a fractional coverage based on a correlation between the number of normalized readings of a region with respect to the number of DNA copies for that region. A minimum inter-sample variability was guaranteed by homogenizing experimental conditions between different samples and genomic regions. CNV calls were classified by DataGenomics based on their credibility using a scoring algorithm that takes into account parameters as log_2_ ratio, event size, proximity, and type of contiguous events. Copy number was calculated upon the sample’s tumor cellularity. CNV plots provided by the platform were manually reviewed to discard possible artifacts and validated by digital PCR or MLPA. The Action OncoKitDx allowed the analysis of CNVs regarding genes included in the panel as well as the study of loss of heterozygosity with a neutral copy number (Copy-neutral LOH) by means of a SNP array evenly distributed throughout the genome.

For structural variant analysis, probes were designed on intronic regions with high probability of presenting fusions. This methodology enabled the identification of any rearrangement in all covered regions. The detection was performed using the BAM alignment file and LUMPY software version 0.2.13, identifying two types of readings surrounding structural events: split reads and discordant read pairs. Distance between discordant read pairs and presence of split reads was used to detect the type of structural event and fusion breakpoint. In combination, they both allowed fusion identification, in addition to other types of large rearrangements, such as deletions or duplications. Artifacts were discarded by quality score assigned by DataGenomics based on split and mate split reads coverage.

For MSI analysis, a panel was designed including 110 microsatellite markers located thorough the genome. A reference was created by determining the allelic combination of the selected regions in a set of microsatellite stable (MSS) samples. Each microsatellite region was thus evaluated by comparison with the model created to categorize it into stable or unstable biomarker. A coverage threshold determined valid results, setting a quality criterion of a minimum of 99 valid markers to analyze a sample’s MSI. The instability fraction is the proportion of unstable markers among the total of valid markers. Fractions between 0 and 0.17 are related to stable samples, from 0.21 to 0.30 correspond to low instability, and above 0.31 are associated to high instability. Fractions between 0.18 and 0.20 give place to inconclusive results.

### 2.6. Variant Functional and Clinical Classification

Mutation analysis was carried out using DataGenomics. This platform enables curation by automatic variant calling and annotation, and allows their prioritization based on quality criteria and clinical relevance. Standard filters were applied to deprioritize variants in order to ensure high-confidence calls based on: (i) technical quality (e.g., coverage ≤ 20, allele frequency in tumor sample ≤ 0.05); (ii) evidence in the internal database (i.e., artifact or benign categorization); (iii) benignity likelihood based on: population frequency ≥ 0.02 in the GnomAD database, and protein effect (e.g., exonic synonymous or intronic non-splicing variants). All remaining variants were submitted for manual review and validation with the Integrative Genomics Viewer (IGV; The Broad Institute, Cambridge, MA, USA).

After variant assessment, functional classification of variants as either pathogenic, likely pathogenic, “variant of unknown significance”, likely benign or benign was performed following the ACMG (American College of Medical Genetics and Genomics) guidelines. Supporting pathogenicity evidence included COSMIC, ClinVar, Varsome, clinical guidelines (NCCN, ESMO), clinical trials, and scientific literature review. Clinical relevance of likely pathogenic and pathogenic variants was automatically reported by Data Genomics, determined by means of the four-tiered ACMG/AMP (Association for Molecular Pathology) guideline system recommended by the Commission of Personalized Medicine (ComPerMed). Variants were classified for their implications in diagnosis (D), prognosis (P), resistance to treatment (R), or therapy (T), indicating the robustness of the available evidence depending on whether it is recommended by clinical guidelines such as SEOM, NCCN (tier I) or if it is widely supported by the literature and databases (tier II).

## 3. Results

### 3.1. Sequencing Performance

The mean Q30 value of the sequencing runs was 90.7%, and 88.4% the average percentage of clusters passing filter, always within the density range recommended by the manufacturer of the sequencing equipment used. The assay achieved an average coverage of 296× after analysis of UMIs. A correct uniformity was obtained with 97.43% of the bases covered at >20% of the average coverage and 71.01% were covered at a depth of 100×.

### 3.2. Analytical Sensitivity and Specificity

We validated the Action OncoKitDx’s accuracy by analytical sensitivity and specificity determination. SNVs and indels were assessed through seven clinical samples. Mutations were confirmed on an amplicon-based mini panel built on the design of other already validated and marketable panels such as the Colorectal OncoKitDx, TP53 OncoKitDx, and BRCA Plus OncoKitDx. Two commercial peripheral blood samples from Coriell Institute including different known variants were diluted at 1:10 with a negative control. Mutations were screened and detected by the Action OncoKitDx. Variants with an allelic fraction above the 5% LOD and located within the panel’s target regions were detected with a mean sensitivity of 100% and specificity of 99%. To support the panel’s accuracy, a reference material from Horizon Dx with over 300 variants at different allelic frequencies, OncoSpan gDNA, was also sequenced. All 70 mutations matching genes included in the panel were detected with similar allelic frequencies.

For the CNV analysis, as well as for the establishment of its LOD, 616 CNV calls including genes in the Action OncoKitDx panel were evaluated among 10 samples previously analyzed with a different NGS panel (Paediatric OncoPanel Dx) or another technique (MLPA, dPCR). Score classification was validated with a 100% concordance between the known variants and their categorization into High-Score.

Nine known structural rearrangements were screened in five samples previously analyzed by real-time PCR or Sanger sequencing. Concordance of results determined a 100% sensitivity and specificity over 99.9%.

The MSI analysis relied on 45 pre-analyzed samples by standard fragment analysis, from which 30 were correctly hybridized and met the quality criteria. Two samples were excluded for not reaching a DIN value higher than 1. A 100% sensitivity and 95% specificity were obtained, with 27 out of 28 concordant cases.

Among six samples, 8 out of all the pharmacogenetic positions included in the Action OncoKitDx were validated by PCR and Sanger sequencing. All 32 positions were confirmed with 100% analytical sensitivity and specificity greater than 99.9%.

### 3.3. Limit-of-Detection

Despite the Action OncoKitDx’s capacity of detecting minor allelic fraction mutations, a 5% LOD was set for SNVs and structural variants, since a considerable proportion of false negatives would be obtained below this threshold. Upon analysis of previously employed samples, the 5% LOD was validated and the CNV detection limit was determined.

**Figure 1:**
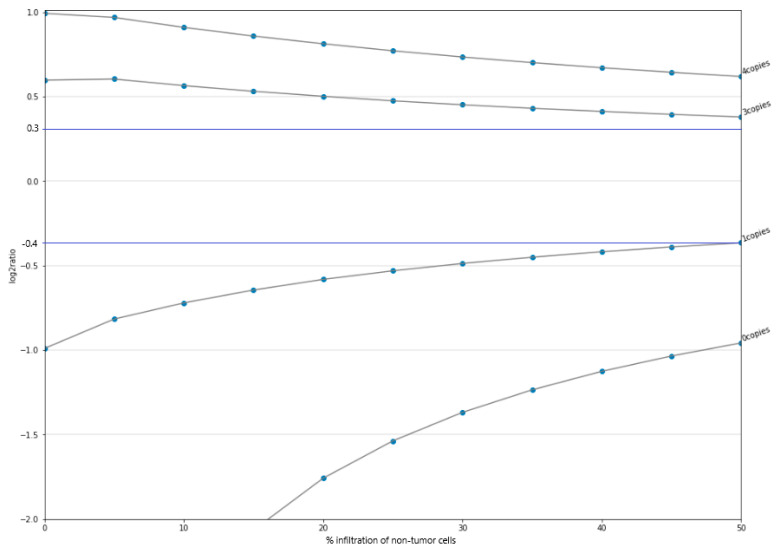
Log_2_ ratio variation of the CNV event according to the number of copies versus percentage of infiltration of non-tumor cells in the tumor sample. Beyond 0.3 (top blue line) for insertions (i.e., number of copies > 2) and below −0.4 (bottom blue line) for deletions (i.e., number of copies < 2) CNVs are detected.

A sample with two know point mutations was diluted in a negative control of similar genomic quality to obtain a 5% allelic frequency. Among three aliquots sequenced, both variants were detected at a frequency close to the expected VAF. For CNVs, graphic representation of the log2ratio variation in four tumor specimens with respect to their percentage of infiltration of non-tumor cells in the sample (Figure 1) allowed the establishment of a threshold ensuring correct gain and loss copy number detection. The LOD called was 3 copies for insertions and ≤1 copy for deletions in samples with up to 50% infiltration with non-tumor cells. However, in samples with a DIN <3 and a non-tumor cell infiltration fraction greater than 50%, the LOD may be decreased.

### 3.4. Repeatability and Reproducibility

To assess repeatability (intra-run), one diluted sample was sequenced by triplicate in one run. Beyond evaluating that a known mutation at a 5% VAF was detected in all three aliquots with a variation coefficient lower than 25%, concordance of results was observed for all variants detected. Among 118 point mutations, 117 were reported in all three, giving a concordance of 99.15%. One discordant variant was excluded from the analysis for being likely an artifact due to its proximity to an homopolymer. Excluding from the analysis CNVs of size less than 0.5 Kb or SCORE lower than or equal to 8, and those present on sex chromosomes X and Y for possible imbalance, results were concordant in all three replicates analyzed. For large rearrangements, poor quality events and those with coverage lower than 10 were excluded from the analysis. In all three samples, one deletion was detected, whose coverage and mate and split readings were consistent in the three samples. MSI was determined, resulting in a MSS fraction 100% concordant among the samples. All the pharmacogenetic positions were studied and correctly detected in the three samples.

Reproducibility (inter-run) was evaluated by analysis of three samples in two different sequencing runs, changing technicians and reagent batches as far as possible. All point mutations included in the regions covered by the Action OncoKitDx with a frequency greater than 5% and filtered by quality PASS and d100 by means of the Data Genomics Platform were considered. After excluding five variants due to their proximity to homopolymers, global concordance of the reproducibility results was 98.3%. Low and medium quality CNVs were excluded, as well as events of size lower than 0.5 Kb. Only one sample had a high-quality event, which was found in both duplicates. Three fusions were detected, each one in a different sample and were coherent in both duplicates. The three selected samples were stable for microsatellites and results were reproducible in all of them. All the pharmacogenetic positions were consistent in both tests for each of the three samples. Finally, results showed a 99.15% repeatability and 98.3% reproducibility for SNV detection, while both values were >99.9% for CNVs, structural variants, MSI and pharmacogenetics. Both reached good robustness, with overall values greater than 99%.

### 3.5. Number of Samples per Run

To calculate the number of samples that can be loaded in a run based on the guarantee of 96.2% of the bases covered at a depth of 100×, a total of 26 samples were analyzed, covering a range of genomic integrities, varying from 1.3 to 8.7.

**Figure 2:**
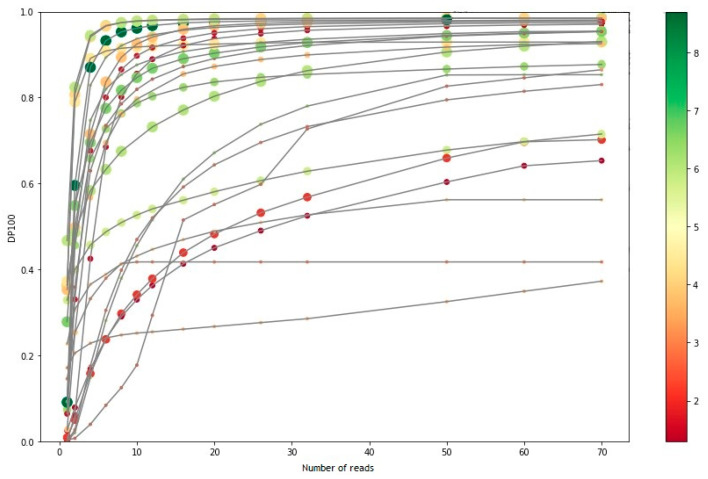
Graphical representation of the DP100 (fraction of bases covered at >100×) of different samples versus their respective number of readings.

As seen in Figure 2, beyond 26 million readings, most samples reached a plateau phase, in which the increase in the number of readings was not reflected in a significant increase in the percentage of bases covered at 100×. The samples that did not reach the plateau were samples of low or medium integrity, from which starting DNA was less than recommended. Since the increase in the number of readings in these samples was not worth the reduction in the number of samples per run, 26 million readings (or 13 million clusters) were established as the minimum number of readings to carry out the Action OncoKitDx sequencing.

Thus, the maximum number of samples recommended per run to guarantee a minimum number of PF clusters of approximately 13 million per sample, which depends on the sequencing kit used, was 13 for NextSeq 500/550 Mid Output v2.5 kit and 32 for NextSeq 500/550 High Output v2.5 kit.

### 3.6. Clinical Feasibility

The validation of the assay’s performance and clinical usefulness in patient samples was performed by comparison with the mutational prevalences from the Cancer Genome Atlas (TCGA) database and by assessment of the clinical potential of the results provided. Total of 14 specimens did not meet the sample or sequencing quality criteria and were excluded from the analysis. A total of 126 tumor samples and 26 controls from 104 patients presenting some of the most frequent cancer types with actionable mutations were included in the different cohorts: breast cancer (*n* = 14), colorectal cancer (*n* = 15), gastrointestinal stromal tumor (*n* = 4), lung cancer (*n* = 19), melanoma (*n* = 19), ovarian cancer (*n* = 10), pancreatic cancer (*n* = 12), and other tumor types (endometrium, cervix, esophagus, larynx, prostate, kidney, thyroid, and bladder, *n* = 11).

**Figure 3:**
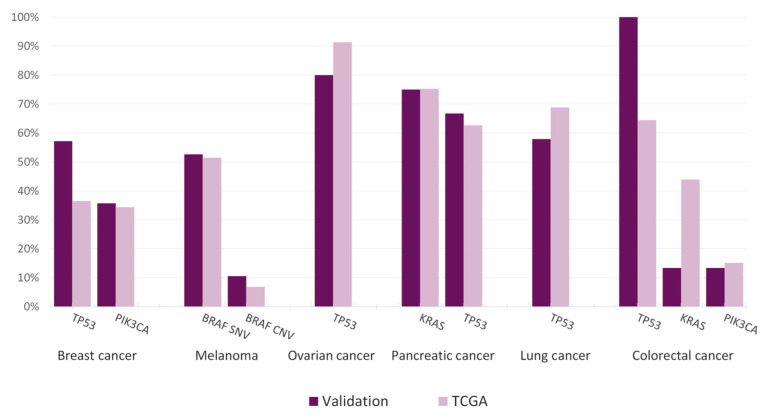
Comparison of the frequencies of the most frequent alterations among different tumor types between the TCGA dataset and the Action OncoKitDx clinical feasibility cohorts (breast cancer, melanoma, ovarian cancer, pancreatic cancer, lung cancer, and colorectal cancer).

The findings from the TCGA project were compared to the mutational landscape obtained within the clinical feasibility cohorts of the main tumor types or with the largest number of specimens. Our results demonstrated good overall correlation, thus allowing cautious extrapolation of the mutational prevalences obtained with the Action OncoKitDx (Figure 3).

In breast cancer, the two main altered genes are *TP53* (36.47%) and *PIK3CA* (34.35%). Results were consistent in our cohort, where frequencies were 57.14% and 35.71% respectively. With respect to melanoma, *BRAF* alteration was the main driver, with comparable SNV and CNV frequencies (52.63% and 10.53% in our cohort, 51.40% and 6.80% in TCGA respectively). In ovarian cancer, *TP53* alterations are almost ubiquitous (91.30% in TCGA), being even more remarkable depending on the subtype. Here we found these aberrations in 80.00% of cases. Two of the main affected genes in pancreatic cancer according to TCGA, *KRAS* (75.27%) and *TP53* (62.64%), were also found in similar proportions in our validation cohort, being 75.00% and 66.67% respectively. In lung cancer the most frequently altered gene is *TP53*, with a global prevalence of 68.77% across the different subtypes. Our clinical feasibility study found a similar frequency of 57.89% in the lung cancer cohort. Finally, the most affected genes in the colorectal cancer TCGA dataset were also observed in our cohort, being *TP53* (64.37%), *KRAS* (43.87%), and *PIK3CA* (15.09%). Despite observing *PIK3CA* at a coherent proportion (13.33%), less consistent results were found for *TP53* (100%) and *KRAS* (13.33%) where frequencies differed from the TCGA dataset.

**Figure 4:**
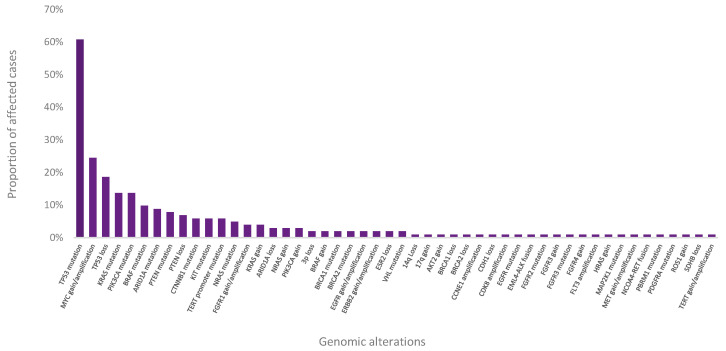
Frequency of the genomic alterations detected.

Pathogenic or likely pathogenic variants were detected in 101 out of 104 patient cases (Figure 4). Among them, actionable mutations with known therapeutic, diagnostic, prognostic, or resistance relevance (TI, TII, DI, DII, PI, PII, RI, RII) were reported for 86 cases.

The *PIK3CA* hotspots (H1047R and E545K) were detected in 5 out of 14 samples from the breast cancer cohort, where beyond being described as an independent negative prognostic factor related to increased tumor aggressiveness, it is suggested to condition resistance to fulvestrant and identifies candidates for alpelisib plus fulvestrant therapy in HR+ and HER2- breast cancers (TI, PII, RII) [3,4,5,6,7,8]. Other investigational biomarkers include different inactivating *TP53* mutations, whose subtype-dependent prognostic value is supported by different studies [9,10].

In colorectal tumors, the G12 *KRAS* mutation detected in 2 out of 15 samples, is predictive for a lack of response to the EGFR inhibitors cetuximab and panitumumab, associated to primary resistance (RI). Despite results on the prognostic value of this biomarker being controversial, studies suggest that *KRAS* activation could condition poor prognosis [11,12]. Nevertheless, its prognostic value seems to depend on the tumor location as it has been observed to indicate a poor prognosis in left-sided patients, whereas right-sided patients did not experience the same effect.

With respect to melanoma, *BRAF* alteration is the main driver although there is a broad range of pathogenic mutations described. However, routine genetic testing is usually performed on exons 11 and 15, where actionable mutations are found. *BRAF* mutations are associated to different recommendations on decision-making according to their location, some related to responsiveness to BRAF and MEK inhibitors (V600E/K) while others have not demonstrated response, as mutations in codons other than V600 in exons 11 or 15. Here we found sensitizing mutations in 9 out of 19 patients (V600E/K) (TI). A T599dup variant was detected, previously identified in cases of thyroid carcinoma, metastatic melanoma, and non-small cell lung cancer but results show contradictory evidence on its sensitivity to inhibitors [13,14]. Moreover, other important alterations are G13 and Q61 in *NRAS* (TII, PI) identified in 3 patients and L576P in *KIT* (TI) in 1 patient. Whereas exon 11 and exon 13 mutations are recognized as sensitizing mutations to KIT inhibition, exon 17 mutations are suggested to have minimal or no sensitivity. The diversity of *KIT* alterations, including amplifications, differing in their sensibility to targeted therapy (e.g., imatinib, sunitinib, nilotinib), make comprehensive genomic profiling essential [15,16,17,18]. *NRAS* mutations are a prognostic factor and its therapeutic impact is under investigation. MEK inhibitors such as bimetinib appear to be a promising treatment option for *NRAS*-mutated melanomas [19,20,21,22].

*BRCA2* (TI), whose biallelic loss is associated to responsiveness to PARP inhibitors and diagnostic relevance with evidence level I, as well as prognostic implications, was altered in 2 out of 10 ovarian cancer patients. Some *PIK3CA* mutations detected (M1043V and H1047R) in 2 samples have approved treatment in other tumoral types, displaying the possibility of potential future therapeutic options.

*KRAS* G12 mutations (DII, PII) were also detected in 10 out of 12 patients of the pancreatic cancer cohort, in which it has diagnostic implications as it can be used to differentiate it from chronic pancreatitis. The presence of mutations in *KRAS* is also associated with lower survival and is more frequent in metastasis than in local disease, for which it is also considered of prognostic relevance [23].

Finally, the most frequently altered gene in lung cancer is *TP53* (PII), unfrequently tested except in case of Li-Fraumeni syndrome suspicion and associated to prognostic relevance [24,25,26]. Missense, nonsense, and splicing mutations were found in 11 out of 19 patients. The analysis also allowed the detection of different characteristic alterations such as the largely described *EGFR* exon 19 deletion (E746_A750del) (TI) which confers sensitivity to EGFR inhibitors carried by 1 patient in this cohort. An infrequent exon 11 *BRAF* mutation (G466V) was detected in 1 patient. Despite activating *BRAF* mutations being very prevalent in lung cancer, the impact on response to *BRAF* inhibitors of changes other than V600 are not well documented and research is necessary.

The assay also enabled the detection of a *EML4-ALK* fusion (TI, RI) in a lung cancer patient. This characteristic alteration confers sensitivity to ALK inhibitors, where alectinib has demonstrated better efficacy than crizotinib, and resistance to EGFR TKIs. A less frequent fusion in colorectal cancer was also revealed. Whereas the *NCOA4-RET* fusion oncogene (TII, PII) found in 1 colorectal cancer patient has been described in this tumor type, it is not usually tested. This alteration could condition a worse prognosis and grant access to RET TKIs in clinical trials [27].

Some relevant copy number variants have been reported in the different cohorts. In breast cancer, *FGFR1* amplification represents the most frequent genomic aberration, mainly observed in the HR+/HER2- subtype [28,29,30]. *FGFR1* amplification is correlated with lower overall survival in HR+ breast cancer [28]. Likewise, an association between *FGFR1* amplification and resistance to endocrine therapy has been described, demonstrating a worse long-term metastasis-free survival in tumors that overexpress *FGFR1*. More recently, *FGFR1* amplification has been suggested as a predictive marker for lack of efficacy of CDK4 and CDK6 inhibitors. This alteration was not only found in 1 breast cancer patient, but also in 3 lung cancers, where despite being a frequent event particularly present in squamous cell carcinoma, no clinical value has been associated. Other amplifications with potential clinical implications in investigation were reported, as *MYC* in 9 melanomas, where it is an important negative prognostic factor, and *EGFR* in 1 breast cancer patient, present in up to 78% of triple negative cases associated with a worse prognosis and more aggressive metastases [31,32]. *VHL* inactivation, either by mutation or 3p deletion, was reported in 2 renal cell carcinomas. These tumors may be sensitive to VEGF receptor-targeted inhibitors and in cases where this gene is the only driver, the occurrence of metastasis is infrequent, while multiple drivers are associated with a higher probability of metastasis [33].

Off-target copy number variants were also identified. *CCNE1* amplification is frequently present in high-grade serous ovarian cancer where it has a certain diagnostic value as it is generally mutually excluding with *BRCA* inactivation. This alteration was found in 1 patient of the ovarian cancer cohort [34,35].

Two MSI-H tumors were identified in the colorectal cancer cohort, where it is a major carcinogenetic pathway, and two MSI-L tumors were found among the renal cell and ovarian cancer groups. The MSI-H reflects a tumor mutator phenotype owed to Mismatch Repair deficiencies (dMMR) [36]. Despite MSI-H being present in most solid tumor types, its prevalence is highly variable. This phenotype, observed in glioblastoma, NSCLC, esophageal, breast, and ovarian cancer is relatively common in CRC, endometrial, and gastric cancer [37,38].

Among the three methods available to determine the MSI-H/dMMR status, two are currently in clinical use: PCR to detect MSI-H and IHC to detect dMMR. These tests are widely used as a screening tool for the detection of Lynch syndrome and the evaluation of prognosis in patients with colorectal cancer. NGS of specific gene panels or whole exome sequencing is emerging as a potentially more analytically specific and sensitive method than PCR or IHC for determining MSI status [39,40]. Whereas traditional MSI testing relies on immunohistochemistry or fragment analysis of at least five microsatellites, 110 microsatellite markers are simultaneously evaluated by Action OncoKitDx with a 99% correlation with the Bethesda panel.

The detection of MSI-H in the colorectal cancer patient could have diagnostic implications since it indicates suspicion of Lynch syndrome (LS), and is a prognostic factor of better survival outcome [41]. Furthermore, while different predictive biomarkers for immunotherapy have been investigated, including PD-L1, MSI/dMMR, and tumor mutational burden (TMB), MSI is currently the only pan-tumor biomarker for an approved oncological treatment. MSI-H in the colorectal tumor could thus provide access to the patient to the administration of Nivolumab or Pembrolizumab approved by the FDA or to clinical trials.

With respect to pharmacogenetics, 71.74% of the cases carried a clinically relevant SNP, up to current knowledge affecting response to different oncologic medications with a level of evidence of 2A or superior, according to the PharmGKB The Pharmacogenomics Knowledgebase (PharmGKB).

## 4. Discussion

Action OncoKitDx, a hybrid capture NGS-based panel indicated in the study of adult solid tumors, was designed to fulfil the actual need of an integral analysis of all biomarkers related to available targeted treatments on the market, approved by international organizations such as the FDA or the EMA, or providing an alternative in recruiting clinical trials. The assay also studies genetic alterations with implications in prognosis, diagnosis, and resistance monitoring with robust and investigational evidence. For this purpose, somatic and germline alterations are analyzed across the complete sequence of 50 genes, 9 fusion genes, 110 microsatellite markers, and 10 pharmacogenetic SNPs.

In this study, we have validated analytical and clinical performance of the Action OncoKitDx in FFPE solid tumor samples, using a protocol that integrates the highly sensitive capture of regions of interest with hybridization probes, with the technique of molecular barcoding of each DNA fragment with a single adapter for high-performance massive sequencing (NGS). This type of protocol allows, during bioinformatic analysis, to carry out the efficient elimination of sequencing and PCR duplicates. The Action OncoKitDx demonstrated a 100% sensitivity for all the biomarkers analyzed. Specificity was adequate, with an overall value of 98.4%, being 95% for the MSI determination and greater than 99% for SNVs, structural variants, and pharmacogenetics. CNVs met the acceptance criterion of 100% concordance assessed by means of confirmation of variants detected with Action OncoKitDx through other methodologies (MLPA, dPCR, NGS panel). Results obtained highlight the robustness of the assay, with overall repeatability and reproducibility values greater than 99%, being 99.15% and 98.3% respectively for SNV detection, while both exceeded 99.9% for CNVs, structural variants, MSI, and pharmacogenetics.

The assay enabled detection of variants in low-quality DNA samples as well as low allelic frequency mutations. In fact, the limit of detection set at 5% for SNVs, CNVs, and structural variants ensured detection in reference standards and clinical material in more than 90% of cases, thus meeting the acceptance criteria. For CNVs, the established LOD was 3 copies for insertions and 1 copy for deletions, in samples with up to 50% infiltration with non-tumor cells. Similar assays designed for the detection of single-nucleotide variants, copy number alterations, and structural variants have been validated showing comparable results, thus supporting the analytical performance of the Action OncoKitDx [42].

The self-developed analytical platform DataGenomics provides an automatic report that includes functional and clinical classification of variants inspired by the Commission of Personalized Medicine (ComPerMed)’s two-level approach, as well as information of clinical relevance for the oncologist’s decision-making provided by the mutational profile of the patient’s tumor. The genetic report finally includes all the relevant information for tailoring personalized patient treatment, including therapies approved by the EMA or FDA agencies and available clinical trials.

Among the 166 samples selected from different hospitals, only 150 (90%) were finally included in the clinical feasibility study cohorts. Low quality of genomic DNA extracted from FFPE tissue specimens is one of the main disadvantages of this form of preservation and preparation technique. In order to avoid the risk of introducing analytical errors, sequencings not meeting the acceptance criteria were excluded from the validation. A total of 141 different pathogenic variants were detected across the sample cohorts, corresponding to 97% of the cases. Despite the limited number of samples in our clinical feasibility cohorts, comparison with the TCGA data showed consistency with the tumor types with the largest number of specimens. The mutational landscape was coherent, even detecting the most frequent mutations at similar prevalences.

Comprehensive molecular profiling has demonstrated significant advantages over the standard practices as individual gene or hotspot testing, or small gene panels used for the recognition of the main mutations with approved therapies. The approach used by NGS-based panels such as the Action OncoKitDx, enable detection of less frequent actionable alterations, mutations with no associated treatment but influence on prognosis, resistance to therapy or diagnosis, or can even provide clinically relevant knowledge on pathogenic variants potentially becoming future therapeutic targets that could identify patients eligible for clinical trials. Among the 150 different pathogenic variants identified, 82 presented clinical significance in their respective tumor types according to the four-tiered ACMG/AMP guideline system, allowing guidance in treatment strategy in 82.7% of cases.

Extensive genomic profiling has proven better reliability in identifying colorectal patients with resistance-associated *KRAS* alterations, melanoma patients with responsive BRAF mutations, lung cancer patients with therapy-associated *ALK* rearrangements, and ovarian cancer patients with somatic *BRCA1/2* alterations who may benefit from PARP inhibitors, than the respective traditional approaches [43,44,45,46]. This clinical feasibility study consistently detected not only non-hotspot actionable alterations who may identify patients benefiting from associated therapies that would not be indicated otherwise, but also all deleterious mutations in the codifying region of the targeted genes.

Besides clinically relevant SNVs, the assay identified different actionable biomarkers, including large and focal CNVs, fusions, MSI-H tumors and, in more than half of the cases, pharmacogenetic genotype-phenotype associations related to response to oncological drugs. MSI-H determination regardless of the tumor type, potentially provides therapeutic options to cancer types where microsatellite instability status would not be routinely determined owing to low incidence. In the research for identification of the most appropriate predictive biomarkers for immunotherapy, TMB and MSI evaluation are emerging as more reliable methods than PDL–1 expression detection, currently used as indication criterion for administration of some checkpoint inhibitors [47,48,49,50]. MSI has become the first pan-cancer predictive biomarker for an FDA-approved therapy (pembrolizumab) and provides prognostic guidance in some tumor types.

While integrating the analysis of different genomic alterations in a single test, the Action OncoKitDx provided a cost-effective, sample and time-saving alternative to the requirement of distinct molecular methodologies, thereby highlighting the importance of the translation of comprehensive genomic profiling into clinical practice.

Among the analytical limitations encountered, the technology used did not allow distinguishing between high-homology regions such as pseudogenes, leading to false positives or negatives. Moreover, below the established quality parameters, specificity, sensitivity, reproducibility, and repeatability of results cannot be guaranteed. NGS technology is not yet considered the “Gold Standard” technique for some types of alterations, so it is recommended, whenever possible, to confirm positive results using complementary and standardized technology. Since this approach has been developed for variants of somatic origin, under suspicion of a germline origin the validation in a non-tumoral sample of the patient (i.e., peripheral blood or normal tissue) is recommended. In case of a clinically interesting finding, specific genetic counselling should be performed due to its hereditary component. Recommendations related to drugs or clinical trials should be taken as a reference for its clinical evaluation and interpretation by the oncologist, in an integrated manner, together with the rest of the patient’s clinical information and results from complementary analytical or imaging tests.

Taken together, our results confirmed compliance of the acceptance criteria established for each analytical parameter, thus validating the suitability of the Action OncoKitDx for the identification of somatic mutations in FFPE solid tumor samples. Herein we validate that the present assay allows the sensitive and specific simultaneous analysis of different actionable alterations and that the technical protocol and the bioinformatic pipeline of the Action OncoKitDx are repeatable and reproducible. Our experience from the implementation of the Action OncoKitDx in the clinical setting has shown great advantage over traditional routine genetic testing approaches. The assay efficiently reports the main driver events and generates valuable information for clinical decision-making.

## Data Availability

The data presented in this study are available within the article.
